# MULTICHANNEL INTRALUMINAL IMPEDANCE MAY BE USEFUL TO IDENTIFY CLINICALLY RELEVANT ESOPHAGOGASTRIC JUNCTION OUTFLOW OBSTRUCTION

**DOI:** 10.1590/S0004-2803.24612025-101

**Published:** 2026-07-20

**Authors:** Felipe Nelson Mendonça, Ricardo Brandt de Oliveira

**Affiliations:** * Universidade de São Paulo, Faculdade de Medicina de Ribeirão Preto, Unidade de Fisiologia GI, Divisão de Gastroenterologia, Ribeirão Preto, SP, Brasil

**Keywords:** Manometry, esophagogastric junction, electrical impedance, esophageal motility disorders, radiography, Manometria, junção esofagogástrica, impedância elétrica, distúrbios da motilidade esofágica, radiografia

## Abstract

**Background:**

A clinically relevant conclusive diagnosis of esophagogastric junction outflow obstruction (EGJOO) requires a manometric diagnosis, relevant symptoms and adjunct exams supporting outflow obstruction. Multichannel intraluminal impedance measurement provides an evaluation of esophageal clearance.

**Objective:**

This study aimed to evaluate the effectiveness of multichannel intraluminal impedance in providing supportive evidence of outflow obstruction and differentiating between severe and mild cases in patients suspected of having esophagogastric junction outflow obstruction.

**Methods:**

A cohort of fifty-nine patients presenting with Abnormal Esophagogastric Junction Relaxation (AEJR), as classified by Chicago Classification version 4.0, was selected from a retrospective, single-center study. Their data from high-resolution impedance manometry were reviewed and compared with clinical and radiologic data. Patients were classified as highly symptomatic (AEJR-HS) or moderately/mildly symptomatic (AEJR-MS) using the Eckardt score.

**Results:**

AEJR-HS was identified in 28 patients and AEJR-MS in 24. Incomplete liquid clearance by multichannel intraluminal impedance demonstrated a strong association with radiological incomplete esophageal clearance (OR: 14.08, 95%CI 2.93 - 87.60, *P*<0.001) and high diagnostic accuracy (93.2%) for EGJOO CC v4.0. Forty percent or more incomplete liquid clearance by multichannel intraluminal impedance during supine swallows showed good accuracy in separating AEJR-HS from AEJR-MS with 75% sensitivity and 83% specificity (AUC=0.825; 95%CI 0.708-0.941, *P*<0.001).

**Conclusion:**

Multichannel intraluminal impedance closely matches esophageal radiography in identifying severe obstructive symptoms and incomplete clearance in AERJ patients and may serve as an alternative to radiography in confirming EGJOO v4.0 in patients with manometric EGJOO.

## HIGHLIGHTS

• Multichannel intraluminal impedance (MII) demonstrates high diagnostic accuracy for identifying patients with clinically relevant abnormal esophagogastric junction relaxation and can distinguish between severe and mild symptomatic cases;

• MII findings of incomplete liquid clearance in supine position strongly associated with radiologic evidence of esophageal contrast stasis, suggesting MII can serve as a reliable alternative to radiography in evaluating esophageal obstruction;

• The use of manometry with MII improves the clinical evaluation of patients with suspected esophagogastric junction outflow obstruction.

## RESUMO

**Contexto:**

O diagnóstico conclusivo da obstrução ao fluxo da saída da junção esofagogástrica (OFJEG) requer o diagnóstico manométrico, sintomas relevantes e testes adjuntos que confirmem a obstrução da junção esofagogástrica. A impedância intraluminal é um método que avalia o clareamento esofágico.

**Objetivo:**

Este estudo tem o objetivo de avaliar a eficácia da impedância intraluminal em fornecer evidência da obstrução ao fluxo esofageano e diferenciar os pacientes com sintomas leves de pacientes com sintomas intensos suspeitos de terem obstrução ao fluxo esofagogástrico.

**Métodos:**

De uma coorte retrospectiva de um centro terciário de referência brasileiro, 59 pacientes apresentando relaxamento anormal da junção esofagogástrica com peristalse presente (RAJEG), conforme a Classificação de Chicago 4.0 (CC v4.0) foram selecionados. Os resultados dos seus exames de manometria de alta resolução com impedância foram revisados e comparados com os parâmetros clínicos e radiográficos. Os pacientes foram classificados em “Muito Sintomáticos” (RAJEG-MS) ou “Pouco Sintomáticos” (RAJEG-PS) conforme o Escore de Eckardt.

**Resultados:**

Vinte e oito pacientes foram classificados como RAJEG-MS e 24 como RAJEG-PS. O clareamento esofágico incompleto pela impedância demonstrou uma forte associação com a estase na radiografia (OR: 14.08, 95%CI 2.93 - 87.60, *P*<0.001) e boa acurácia diagnóstica (93.2%) para OFJEG CC v4.0. Quarenta porcento ou mais deglutições supinas com clareamento esofágico incompleto pela impedância demonstrou boa acurárica em diferenciar pacientes RAJEG-MS de RAJEG-PS com 75% de sensibilidade e 83% de especificidade (AUC=0.825; 95%IC 0.708-0.941, *P*<0.001).

**Conclusão:**

A impedância intraluminal tem grande concordância com a radiografia esofágica em identificar sintomas obstrutivos intensos e pode ser uma ferramenta útil a para confirmar a OFJEG CCv4.0.

## INTRODUCTION

The combination of impaired lower esophageal sphincter (LES) relaxation (defined as an abnormally high median IRP according to the Chicago Classification), and absent peristalsis is the manometric hallmark for achalasia, a well-defined clinical entity^1^. Differently, the clinical significance of incomplete LES relaxation with preserved esophageal peristalsis has been elusive^2^ ^,^ ^3^ ^,^ ^4^. With the advent of high-resolution esophageal manometry (HREM), the combination of impairment of LES relaxation in the supine position has become frequently detected, leading to its inclusion in the Chicago Classification (CC) as a manometric entity under the name of Esophagogastric Junction Outflow Obstruction^5^.

The widespread use of HREM has made it clear that EGJOO, based in the original acquisition protocol using solely supine swallows, correlates poorly with relevant obstructive symptoms^2^ ^,^ ^3^ ^,^ ^6^. Many patients show no symptoms of outflow obstruction, with some experiencing transient or no symptoms at all^2^ ^,^ ^3^ ^,^ ^4^ ^,^ ^7^. These findings have led to the investigation of additional procedures to enhance the diagnostic accuracy of EGJOO.

An important outcome of this effort revealed that a significantly high IRP in both supine and sitting positions can eliminate most, but not all, EGJOO cases that lack clinical relevance^5^ ^,^ ^8^ ^,^ ^9^ ^-^ ^11^. These observations have prompted further pursuit of complementary methods to enhance the diagnostic accuracy of EGJOO and evidence was produced suggesting that elevated intrabolus pressure (IBP) and provocative manometric tests like Multiple Rapid Swallows (MRS), Solid Swallows (SS), and Rapid Drink Challenge (RDC) may help identify significant cases of EGJOO^12^ ^,^ ^16^. In this context, CC v4.0 has refined the criteria for diagnosing EGJOO to more accurately identify patients who might benefit from procedures addressing EGJ obstruction^6^. Specifically, an EGJOO diagnosis should be considered for patients presenting with dysphagia or chest pain and exhibiting abnormal IRP in both supine and upright positions with elevated intrabolus pressure^6^. In addition, MRS, RDC, and SS are suggested as supportive measures to increase diagnostic confidence, and EGJOO’s clinical relevance should be validated with a complementary exam like barium esophagography or Functional Lumen Imaging Probe (FLIP) to confirm EGJ obstruction.

Multichannel intraluminal impedance (MII), a radiation-free technique, has been proposed for evaluation of bolus transit and esophageal clearance^17^ ^,^ ^18^. MII is closely correlated with barium radiography for evaluating esophageal emptying and is effective in demonstrating incomplete bolus clearance in patients with supine IRP elevation and normal peristalsis^19^ ^-^ ^21^. Arguably, MII can be included in the diagnostic approach for EGJOO, especially as impedance data can be collected simultaneously with manometric data.

While dysphagia is crucial for diagnosing EGJOO^6^, few studies have analyzed the diagnostic yield of EGJOO for severe obstructive symptoms in comparison to individual manometric parameters, radiological assessments, and impedance measurements^2^ ^,^ ^8^ ^,^ ^15^.

We hypothesize that MII could detect incomplete esophageal clearance and assess obstructive symptom severity in patients with impaired LES relaxation. Additionally, evaluating the CC v4.0 criteria for EGJOO and other manometric criteria, as well as MII and radiology on patients with impaired IRP relaxation stratified by their clinical relevance, could enhance the identification of those who may benefit from invasive therapy.

The objectives of the current research were first, to evaluate the capability of MII in detecting incomplete esophageal clearance in abnormal esophagogastric junction relaxation (AEJR) patients, second, to compared MII, radiology, manometric variables, and EGJOO diagnosis in their ability to distinguish AEJR patients with severe symptoms from those with mild to moderate symptoms.

## METHODS

### Study design

**FIGURE 1:**
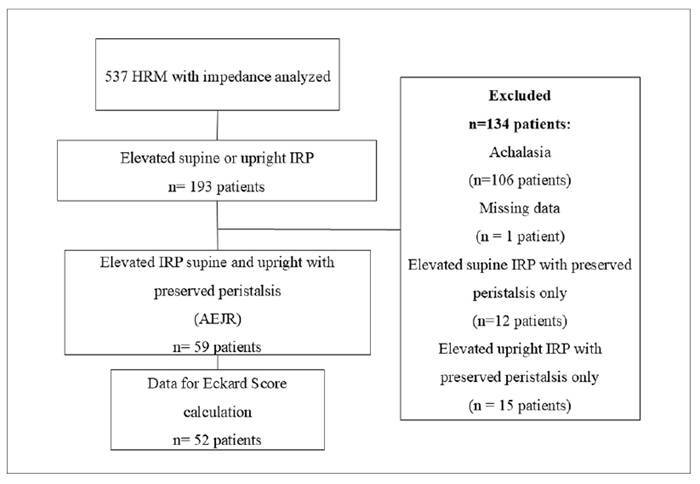
Patients’ selection criteria flow chart.

This is a retrospective single-center study. All results of HREM studies performed in our lab from January 2011 to January 2022 were reviewed to select our study group. The criteria for inclusion were: (1) elevated median single swallows IRP values in both supine and upright positions (above 23.5mmHg in supine and 15mmHg in upright) according to published normal values for HREM obtained with Sandhill HREM^22^ system and (2) evidence of any preserved peristalsis. Combined (1) and (2) criteria define what is herein called Abnormal Esophagogastric Junction Relaxation (AEJR). Both structural (mechanical or anatomical alteration of esophagus, like hiatal hernia, esophageal diverticulum, malignancy, strictures; or previous esophagogastric surgery) and functional AEJR (absent structural disorder in X-Ray and/or endoscopy) were included in this study. All HREM recordings were meticulously reviewed and patients with inconclusive achalasia diagnoses or incomplete HREM with impedance protocols (including MRS studies or those conducted without sitting position) were excluded. The patient selection flow chart is presented in Figure 1. Thirty-one healthy subjects with no upper gastrointestinal complaints or surgery history underwent the same esophageal HREM protocol as the control group. This study was reviewed and approved by the hospital Institutional Ethics Committee.

### Acquisition protocol

HREM was performed using a 32-channel Unisensor solid-state catheter system with 1-cm intervals and 16 impedance sensors each 2-cm intervals (InsiGHT™ HREM, Sandhill Scientific Inc., Highlands Ranch, CO, USA). Prior to commencing the examination, calibration at 0 and 100 mmHg was performed using externally applied pressure and impedance recording. This procedure involved immersing the catheter into a water-filled tube and verifying the impedance drop across all sensors. The catheter was passed through a nostril and positioned to record from the hypopharynx to the stomach, with at least three gastric sensors.

In all patients, standard protocol was performed with (1) a baseline recording at least 30s swallow-free; (2) 10 5-mL saline swallows in upright position; (3) 10 5-mL saline swallows in supine position; (4) two Multiple Rapid Swallows (MRS) with five 2-mL successive saline swallows spaced 2-3s apart in supine position; (5) and two sets of MRS in upright position. Thermal compensation was applied at the end of the recording.

### Analysis of high resolution manometry recordings

The analysis consisted of: (1) Visual inspection and classification of esophageal body motility pattern and EGJ morphology; (2) Measurement of the following: integrated relaxation pressure (IRP), supine resting LES pressure (LESP), esophagogastric junction contractile integral (EGJ-CI), distal contractile integral (DCI), distal latency (DL), intrabolus pressure (IBP). IRP obtained during provocative tests were referred to as MRS-IRP in Multiple Rapid Swallows. MRS-IRP was measured from the first swallow until the pressure wave of the esophageal body according to Kushnir et al.^23^. As Sandhill’s software lacks an intrabolus pressure (IBP) measurement tool, we used the Mean Respiratory Pressure from the channel 1 cm above LES during 3-seconds peak pressure zone within swallowing LES relaxation time.

### Analysis of multichannel intraluminal impedance recordings

Bolus clearance after each swallow was considered complete if bolus entry (ie, 50% decrease in impedance from baseline to nadir) occurred at the 20-cm channel, and bolus exit (ie, return to 50% impedance point) occurred at the 15-cm, 10-cm, and 5-cm channels and incomplete if bolus exit was not identified at any of the 3 distal impedance measurement sites. In BioView Analysis software (Sandhill Scientific, Highlands Ranch, CO, USA), the impedance sensors used were channels 5, 7, 9 and 12, of which are 18, 14, 10 and 4 cm above EGJ respectively. The proportion of swallows with complete liquid bolus clearance of each subject was registered in percentage and liquid bolus clearance was considered complete when at least 80% of the swallows were complete^17^.

### Clinical data evaluation

We revised the medical records of all patients to retrieve clinical data, laboratory exams and medical imaging studies carried out during investigation, therapy (if employed) and post-therapy outcome. We used the Eckardt Score as measurement for severity of symptoms^24^. Dysphagia, chest pain, and regurgitation were graded from 0 to 3, where 0 was no symptoms, 1 was occasional symptoms, 2 was daily symptoms, and 3 was symptoms with each meal. Weight loss was graded as 0 for no weight loss, 1 for less than 5 kg lost, 2 for 5-10 kg, and 3 for >10 kg. Patients were classified as “Highly Symptomatic” (AEJR-HS) when Eckardt Score (ES) was greater than 3 points and/or dysphagia subscore greater or equal 2 points; and “Mild to Moderately Symptomatic” (AEJR-MS) when ES less or equal than 3 points and dysphagia subscore less than 2 points.

### Barium esophagography

Patients either underwent conventional barium swallows (esophageal serigraphy) with ingestion of 150mL of barium sulphate 100% (1g.mL^-1^) or Timed Barium Esophagography (TBE) with images taken at 10 seconds, 1 minute, and 5 minutes post-ingestion. Incomplete barium clearance was noted if there was any barium column after 1 minute in TBE or retention at the EGJ in serigraphy.

### Statistical analysis

Statistics were presented as mean ± standard deviation and median (25^th^ and 75^th^ percentiles). The Wilcoxon rank sum test was used for manometric, impedance and radiography quantitative variables, and Fisher’s exact test for qualitative variables, with Haldane-Anscombe correction if needed. Multivariate logistic regression predicted symptomatology, with variables selected by Backward statistics when p < 0.20. Receiver Operating Characteristics curves (ROC) determined sensitivity, specificity and accuracy with optimal cutoffs. All *P*-values were two-tailed, with 0.05 as the threshold for significance. Data analysis was conducted using R software version 4.2.2.

### Ethical considerations

This study was reviewed and approved by the Institutional Ethics Committee of University of São Paulo - Ribeirão Preto. A written informed consent was obtained from all patients.

## RESULTS

Of 539 consecutive HREM studies, 59 (5.40%) patients met the criteria for AEJR. The control group consisted of 31 healthy subjects. Records for 52 AEJR patients were suitable for ES calculation (Figure 1). Of these, 28 (53.80%) were “Highly Symptomatic” (AEJR-HS) and 24 (46.20%) “Mild to Moderately Symptomatic” (AEJR-MS). Demographic details are in Table 1.

**TABLE 1:** Demographic, clinical, manometric, impedance and radiographic variables in AEJR, AEJR-HS, AEJR-MS and controls subjects.

Variable	AEJR	AEJR-HS	AEJR-MS	Controls
	n=59	n=28	n=24	n=31
Age				
Mean (SD) Amplitude	57.3±12.4 (30 - 78)*	60.9±10.9 (40 - 78)	55.3±13.0 (34 - 78)	36.3±9.77 (20 - 55)
Female n (%)	44/59 (74.5%)	24/28 (85.7%)	17/24 (70.8%)	19/31 (61.2%)
BMI Mean (SD)	30.9±9.0	28.1±6.9	35.0±10.1	27.2±5.16
Upright IRP (mmHg)	20.2 (16.9, 26.1) * †	22.2 (18.2; 31.6)	18.0 (16.4; 22.2)	6.4 (3.3; 10.7)
Supine IRP	26.5 (24.1; 29.4) *	27.4 (24.8; 29.9) **	24.6 (23.3; 26.9)	13.7 (9.5;16.1)
Supine Resting LES pressure (mmHg)	33.4 (22.1; 42.0)	30.8 (16.4; 41.6)	33.5 (27.2; 40.1)	30.9 (21.4; 44.8)
Supine EGJ-CI (mmHg.cm)	53.2 (31.1; 72.3) *	61.5 (31.1; 80.9)	44.4 (29.8; 73.7)	26.2 (10.2; 39.9)
Upright DCI (mmHg.cm.s)	1861 (856.5; 2922.5) * †	1968.2 (798.8; 3145.5)	1731.5 (915.1; 2839.8)	1204 (670.4; 2005)
Supine DCI	2614 (1887; 3956) *	2363 (1158; 3962.3)	2674 (2207.0; 4149.2)	2283 (1593; 4494)
Upright DL (seconds)	6.1 (5.6; 6.9)	5.9 (5.5; 6.7)	6.5 (5.9; 7.1)	6.5 (5.8; 7.4)
Supine DL	6.6 (5.9; 7.4)	6.3 (5.9; 7.0)	6.8 (5.9; 7.5)	6.8 (6.3; 7.6)
Upright IBP (mmHg)	16 (13; 20) * †	18.7 (15.1; 23.2)**	14.7 (11.7; 16.6)	6.7 (4.5; 9.3)
Supine IBP	19 (16.5; 21.7) *	20 (16.5; 23.2)	17.5 (14.8; 20.0)	12.8 (9.8; 14.7)
Upright IRP-MRS (mmHg)	17.4 (13.4; 21.6) * †	19.7 (14.2; 23.7)	15.3 (12.1; 20.5)	8 (5.5; 9.0)
Supine IRP-MRS	22.0 (18.4; 25.8) *	24.5 (20.0; 28.7) **	20.7 (16.6; 23.3)	12.4 (10; 15.3)
Incomplete liquid clearance				
(% swallows)				
Upright	10% (0; 55%)	35 (7.5; 80%)	10% (0; 32.5%)	-
Supine	20% (0; 60%)*	55% (15; 90%)**	0% (0; 20%)	0% (0; 0%)
Incomplete clearance in radiography	22/44 (50%)♦	19/26 (61.5%)**	3/15 (20%)	-

*P<0.050 comparing AEJR vs Controls. **P<0.050 comparing AEJR-HS vs AEJR-MS. †*P*<0.050 comparing supine vs upright position in AEJR group. ♦44 AEJR patients had radiography but 41 had ES for calculation. IQR: interquartile range. SD: standard-deviation. BMI:body mass index. *P*-values were based on the Wilcoxon rank sum test for quantitative variables and Fisher’s exact test for qualitative variables.

Data of manometric variables are medians with IQR in parenthesis. Data of impedance are medians of percentage of abnormal results with IQR in parenthesis. Data of radiography are the absolute number of abnormal results and percentage in parenthesis.

### Clinical characteristics

AEJR patients were older than controls, but not different regarding gender, weight and BMI. AEJR-HS and AEJR-MS were not different regarding age, gender, weight and BMI (Table 1). Medians ES for AEJR-HS and AEJR-MS were 3.50 (3.00 - 6.00) and 0.50 (0 - 1.25) points respectively. The most common presenting symptoms in AEJR were dysphagia in 40 patients (75.4%), regurgitation in 14 patients (26.4%) and chest pain in 10 patients (18.8%); 12 patients were asymptomatic (22.6%). The drug class most prescribed was proton pump inhibitors to 24 patients (45.2%); 5 (9.4%) were in use of antipsychotics. Six patients (11.3%) kept on smooth muscle relaxants (nifedipine and isosorbide dinitrate) stopped at least seven days before HREM. No patient was a chronic opioid user. The most common comorbidities were arterial hypertension (42.3%) and *Trypanossoma cruzi* positive serology (49.0%). Twelve patients (20.3%) had diabetes mellitus, 16 patients (27.1%) had psychiatric comorbidities of which, anxiety in 8 (50.0%); depression in 10 (62.5%); bipolar disorder in 2 (12.5%) and obsessive-compulsive disorder in 1 (6.25%). Three had rheumatologic diseases (5.0%): one with cutaneous-limited systemic sclerosis; one with systemic erythematous lupus and one with Sjogren syndrome.

Fifty patients (84.7%) underwent upper GI endoscopy, 44 (74.5%) had esophageal radiography. According to X-Ray and/or endoscopic results studies, 20 patients (33.8%) had structural-AEJR: 15 had hiatal hernia, 7 previous fundoplication, 2 previous partial gastrectomy, and one a leiomyoma in distal esophagus. Thirty-four patients (57.6%) with no detected anatomical abnormality in X-Ray or endoscopy had functional-AEJR. Anatomical evaluation was missing in five patients (8.4%).

### Manometric variables

Table 1 shows the manometric variables for AEJR, AEJR-HS, AEJR-MS and control groups. Supine IRP was higher in AEJR-HS patients than it is in AEJR-MS (*P*=0.034). Supine IRP-MRS was higher among AEJR-HS compared with AEJR-MS (*P*=0.005), with no difference in upright IRP-MRS. Upright IBP was higher in AEJR-HS than in AEJR-MS (*P*=0.041).

Forty three AEJR patients (73%) had normal contractility, 8 (13.5%) had Ineffective Esophageal Motility (IEM), 5 (8.5%) Hypercontractility and 3 (5%) Distal Esophageal Spasm (DES). ES in AERJ patients with abnormal contractility (median 3, interquartile range [IQR]: 2-7 points) was significantly higher (*P*=0.034) than in patients with normal contractility (median: 2.0, IQR: 0-3.0 points). Among AERJ patients, ES was significantly higher in presence of IEM (median: 8, IQR: 4-8.5 points; *P*=0.004) and DES (median: 6, IQR: 4.5-6.5 points; *P*=0.043) compared to normal contractility. ES in AERJ with hypercontractility did not differ from AERJ with normal contractility (3, 1-5 points; *P*=0.450). AEJR-HS and AEJR-MS did not differ regarding proportions of abnormal results of IRP, IRP-MRS, IBP as shown in Table 2.

Patients with positive *T. cruzi* serology group had lower LES pressures (*P*=0.008), EGJ-CI (*P*<0.001) and lower supine IBP (*P*=0.028) than the negative serology group. All other clinical, manometric, impedance and radiographic data were not different between both positive and negative serology groups.

### Liquid bolus clearance by impedance in AEJR

**FIGURE 2:**
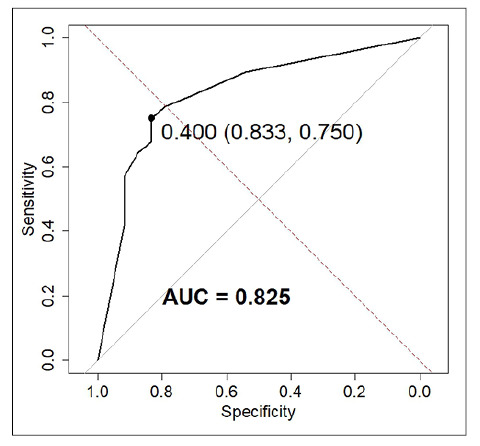
Receiver Operating Characteristic (ROC) analysis of Incomplete Liquid Bolus Clearance in supine MII and patient being highly symptomatic. An optimal cutoff of 40% or more swallows with incomplete liquid bolus clearance had good accuracy in separating AEJR-HS from AEJR-MS.

The proportion of incomplete liquid bolus clearance was significantly higher in patients with AEJR compared to the control group, as presented in Table 1. More patients had incomplete liquid clearance in supine swallow with AEJR-HS than with AERJ-MS (*P*=0.004). A significant association between incomplete liquid bolus clearance in the supine position and patients highly symptomatic was found (Table 2). By ROC analysis, the optimal cutoff of percentage of supine swallows with incomplete liquid bolus clearance for predicting AERJ-HS was 40% (Figure 2), and this cutoff yielded a sensibility of 75% (95%CI 41% - 92%), specificity 83% (95%CI 62% - 99%) and AUC=0.825 (95%CI 0.708-0.941, *P*<0.001).

In patients with AEJR, the proportion of swallows with incomplete liquid bolus clearance was notably higher when esophageal contractile body abnormalities were present (median percentage of swallows 70%, IQR: 25-95%) compared to when these abnormalities were absent (10%, IQR: 0-30%; *P*=0.008). Patients with IEM (median percentage of swallows 75%, IQR: 57.5-92.5%) and DES (median percentage of swallows 70%, IQR: 65-80%) had a higher proportion of swallows with incomplete clearance compared to those with normal contractility (*P*<0.001 for IEM, *P*=0.030 for DES). However, this was not observed in patients with hypercontractility (median percentage of swallows 10%, IQR: 0-100%, *P*=0.604).

### Radiographic variables

Forty-four of 59 (74.5%) AEJR patients had radiography done, in which, 41 of them had ES calculated (69.4%). Incomplete barium clearance was seen in 19 out of 26 exams (73.0%) from the AEJR-HS group and 3 out of 15 exams (20%) from AEJR-MS (Table 1). AEJR-HS was associated with incomplete barium clearance (OR: 10.11; 95%CI 1.96 - 73.10, *P*=0.001), as shown in Table 2.

**TABLE 2:** Proportions of manometric, impedance and radiographic variables with abnormal results in AEJR-HS and AEJR-MS and their association to the AEJR-HS group.

Variable	AEJR-HS	AEJR-MS	***P*-value**	OR (95%CI)
	Abnormal results	Abnormal results		
	n(%)	n(%)		
Upright IBP	22/28 (78.5%)	15/24 (62.5%)	0.233	2.16 (0.55 - 9.12)
Supine IBP	18/28 (64.2%)	12/24 (50%)	0.400	1.77 (0.51 - 6.32)
Upright MRS-IRP	22/28 (78.5%)	18/24 (75%)	0.738	1.45 (0.31 - 7.12)
Supine MRS-IRP	20/28 (71.4%)	12/24 (50%)	0.155	2.45 (0.69 - 9.22)
Upright Incomplete liquid clearance by MII	18/28 (64.2%)	8/24 (33.3%)	0.050	3.5 (0.99 - 13.33)
Supine Incomplete liquid clearance by MII	19/28 (67.8%)	5/24 (20.8%)	<0.001	7.65 (1.96 - 35.4)
Incomplete liquid clearance by radiography	19/26 (73.0%)	3/15 (20%)	0.014	10.11 (1.96 - 73.10)
EGJOO CCv4.0	14/28 (50%)	2/24 (9.0%)	0.002	10.49 (1.95 - 109.12)
EGJOO CCv4.0 using MII instead radiography	13/28 (46.4%)	3/24 (12.5%)	0.014	5.85 (1.29 - 37.66)
Abnormal body contractility	11/28 (39.2%)	5/24 (20.8%)	0.228	2.41 (0.61 - 10.79)

IBP: intrabolus pressure. MRS-IRP: integrated relaxation pressure in multiple rapid swallows. MII: multichannel intraluminal impedance. EGJOO CC v4.0: esophagogastric junction outflow obstruction as Chicago classification version 4.0.

**FIGURE 3:**
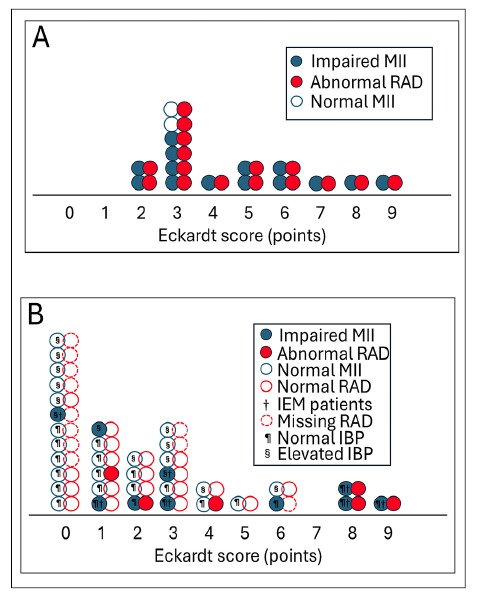
AERJ patients categorized based on their esophageal clearance results, which were evaluated using both impedance and radiology. The scatterplot depicts their ES values. Each intersecting circle pair corresponds to a specific subject. Panel A: sixteen AERJ patients meeting criteria for EGJOO CC v4.0; Panel B: AERJ patients not meeting criteria for EGJOO.

Incomplete barium clearance by radiography showed moderate concordance with incomplete liquid bolus clearance by supine MII (*k*=0.591, *P*<0.001), moderate concordance in upright MII (*k*=0.455, *P*=0.002), and a strong concordance is seen among patients exhibiting severe symptoms (Figure 3A). The distribution of radiography and impedance data of AEJR patients that had not fulfilled EGJOO CC v4.0 criteria is shown in Figure 3B.

The association between incomplete barium clearance observed through radiography and incomplete liquid bolus clearance measured by MII was found to be significant in both supine (OR: 14.08, 95%CI 2.93 - 87.60, *P*<0.001) and upright position (OR: 6.75, 95%CI 1.60 - 33.22, *P*= 0.006).

Using incomplete barium clearance in radiographic analysis to detect outflow obstruction, EGJOO CC v4.0 was identified in 16 patients; using MII supine incomplete clearance, 14 of them would have been diagnosed with EGJOO (Figure 3A). EGJOO defined by using MII to confirm outflow obstruction, predicted EGJOO based on barium stasis with an accuracy rate of 93.2%, a sensitivity of 87.5%, and a specificity of 95.3%. 

### Prediction of symptoms

Multivariate logistic regression showed that males had an 88% lower risk of being highly symptomatic (*P*=0.047). The higher the IRP value during supine MRS (OR: 1.18 (95%CI: 1.04-1.39) and the greater the number of supine swallows with incomplete clearance in impedance (OR: 1.27 (95%CI: 1.04-1.61), the higher the predicted ES (*P*=0.025 and *P*=0.028 respectively).

## DISCUSSION

In a 10-year retrospective study at a single center, we reviewed the clinical, manometric, impedance and radiologic data of 59 patients with abnormal EGJ relaxation by CC v4.0 criteria, namely, preserved esophageal peristalsis and abnormal median IRP in both supine and sitting upright position (hereinafter referred as AEJR). Overall, our findings indicate that MII could be beneficial in the assessment of patients with suspected EGJOO and other esophageal motility disorders.

We observed that MII results during liquid swallows in the upright and supine position align closely with radiology in detecting incomplete esophageal clearance. MII results predicted obstructive symptoms in AEJR patients as accurately as radiology, consistently across all ES values. These findings align with previous studies that indicate a significant correlation between incomplete bolus emptying by MII and symptoms of esophageal motor disorders in EGJOO CC v3.0 patients^20^ ^,^ ^21^ ^,^ ^25^ ^,^ ^26^.

Notably, the association of AEJR-HS with incomplete liquid clearance by MII in supine swallows is stronger than the associations with abnormal results of any individual parameter, including IBP, which assists in diagnosing EGJOO according to CC v4.0.

Our findings indicate that MII not only accurately identifies obstructive symptoms in AEJR but also effectively assesses their severity. Patients with severe symptoms have a higher rate of incomplete liquid clearance compared to those with mild to moderate symptoms. Our study indicates that more supine swallows with incomplete liquid clearance in MII lead to higher ES and that cutoff of 4 or more impaired swallows accurately distinguishes AEJR-HS from AEJR-MS. To our knowledge, no previous study has utilized impedance cutoffs to ascertain clinical relevance in EGJOO. Using MII accurately identified EGJOO versus achalasia, but no threshold was established for differentiation^26^.

CC v4.0 recommends that incomplete esophageal clearance is needed for a conclusive EGJOO diagnosis, obtainable through FLIP or radiology^6^. Our findings indicate that MII can also be used as complementary testing to confirm the diagnosis of EGJOO conclusively. MII may offer certain advantages compared to other methods of outflow obstruction. MII does not involve radiation and can be conducted alongside HRM, enabling simultaneous assessment of liquid bolus transit and esophageal motility through manometry. FLIP is accurate and can be performed during sedated endoscopy, but it is not widely accessible, therefore out of reach for many around the world^27^.

MRS is a maneuver in HREM used to assess contractile reserve^28^ and EGJ deglutitive inhibition^23^. Our study demonstrated that high IRP during MRS in supine position predicted higher ES values in AERJ patients. Recent studies show that IRP measurement during MRS has accuracy like that of RDC in detecting abnormal barium retention^13^ and clinically relevant symptoms^15^.

AEJR patients with IEM were notably symptomatic with a median ES of 8 (IQR: 4-8.5). Some of them failed to meet EGJOO CC v4.0 criteria due to the absence of elevated IBP and, despite being severely symptomatic, could be excluded from treatment consideration. IBP depends on strength of peristalsis^29^ ^-^ ^31^ and in AEJR with IEM patients, peristalsis may be too weak to increase IBP beyond normal levels.

In our study, all DES patients exhibited significant symptoms, which aligns with findings that DES is like type III achalasia^32^ ^-^ ^34^. A recent analysis proposes a new subset of patients with EGJOO and abnormal body motility, termed mixed motility disorder, who respond well to dilation or endoscopic/surgical treatment^35^. Our study observed that patients with AEJR and hypercontractility exhibited less impairment in clearance by impedance and experienced fewer symptoms, comparable to those with normal contractility. In this context, the robust contractility effectively promotes flow across the esophagogastric junction, resulting in more complete liquid clearance and fewer symptoms^14^ ^,^ ^19^ ^,^ ^20^.

This study has several limitations. No validated dysphagia questionnaire was used. The Eckardt score, used to evaluate clinic severity, was originally devised to measure the results of achalasia treatments, but its comparison with more sophisticated, validated scores shows only minor differences in results^36^. ES has demonstrated ease of application and fair reliability for non-achalasia patients^36^ ^,^ ^37^. Since the study involved patients with a high likelihood of EGJOO, our results may not apply to other populations. Our study primarily used esophageal serigraphy to evaluate esophageal clearance, although TBE, the standard for esophageal motor disorders^38^, the serigraphy consistently identified highly symptomatic patients. Of note, serigraphy has been used in other studies to evaluate esophageal clearance^13^ ^,^ ^25^ ^,^ ^39^ ^,^ ^40^. Comparisons between the accuracy of different metrics are limited by small sample sizes, but they provide a proof of concept that encourages further exploration with larger samples, longer follow-up and greater statistical power. Our population study included individuals with positive serology for T. cruzi, who could exhibit atypical results. The main difference between *T. cruzi*-positive and *T. cruzi*-negative patients was the lower basal pressures of lower esophageal sphincter in the positive group, consistent with previous studies on esophageal involvement in Chagas’ disease^41^. Another important fact is to highlight that Chagas’ disease has increasingly become a global health concern, with rising prevalence reported in developed countries^42^ ^,^ ^43^. Given such context, including these patients in studies is both relevant and necessary.

In conclusion, multichannel intraluminal impedance significantly matches with esophageal radiography results regarding esophageal clearance and obstructive symptoms in AERJ. Multichannel intraluminal impedance may serve as an effective radiation-free method for diagnosing EGJOO CC v4.0 in patients with manometric EGJOO, including those with abnormal contractility patterns.

## Data Availability

data-available-upon-request.
